# Supplementary figures and images for: Impact of HLA type, age and chronic viral infection on peripheral T-cell receptor sharing between unrelated individuals
Source: PLoS One. 2021 Aug 30;16(8):e0249484. doi: 10.1371/journal.pone.0249484 (PMC8405014; doi:10.1371/journal.pone.0249484)

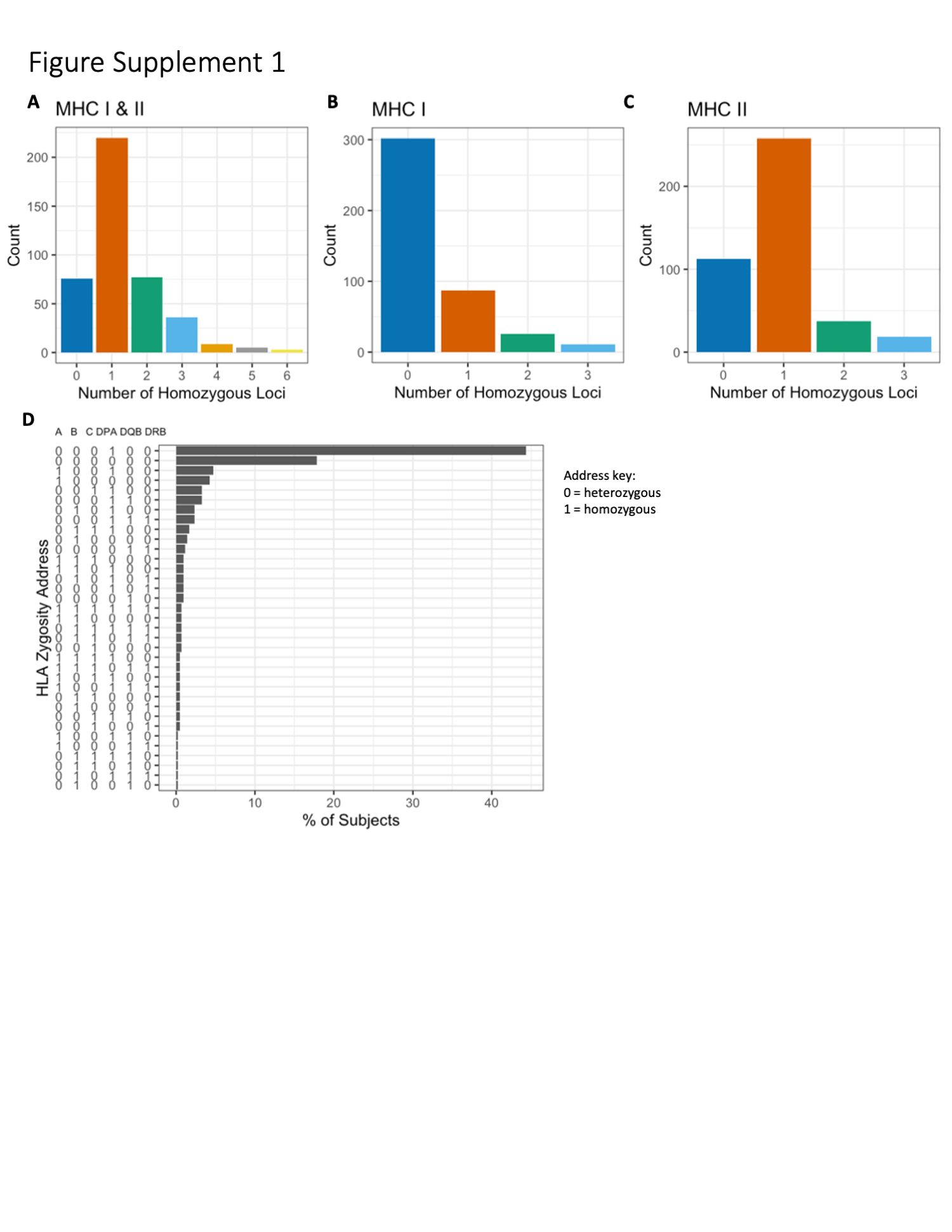

Supplement: S1 Fig — (A) Count of individuals, among the cohort of 426, based on number of homozygous HLA loci. (B) Count of individuals based on number of homozygous HLA class I loci: HLA-A, HLA-B, HLA-C. Most individuals are heterozygous at all HLA Class I loci. (C) Count of individuals based on number of homozygous HLA class II loci: HLA-DPA1, HLA-DQB1, HLA-DRB1. (D) The distribution of individuals with each combination of homozygous loci. 44% of individuals in this cohort are homozygous at HLA-DPA1. (TIF) [file pone.0249484.s001.tif]

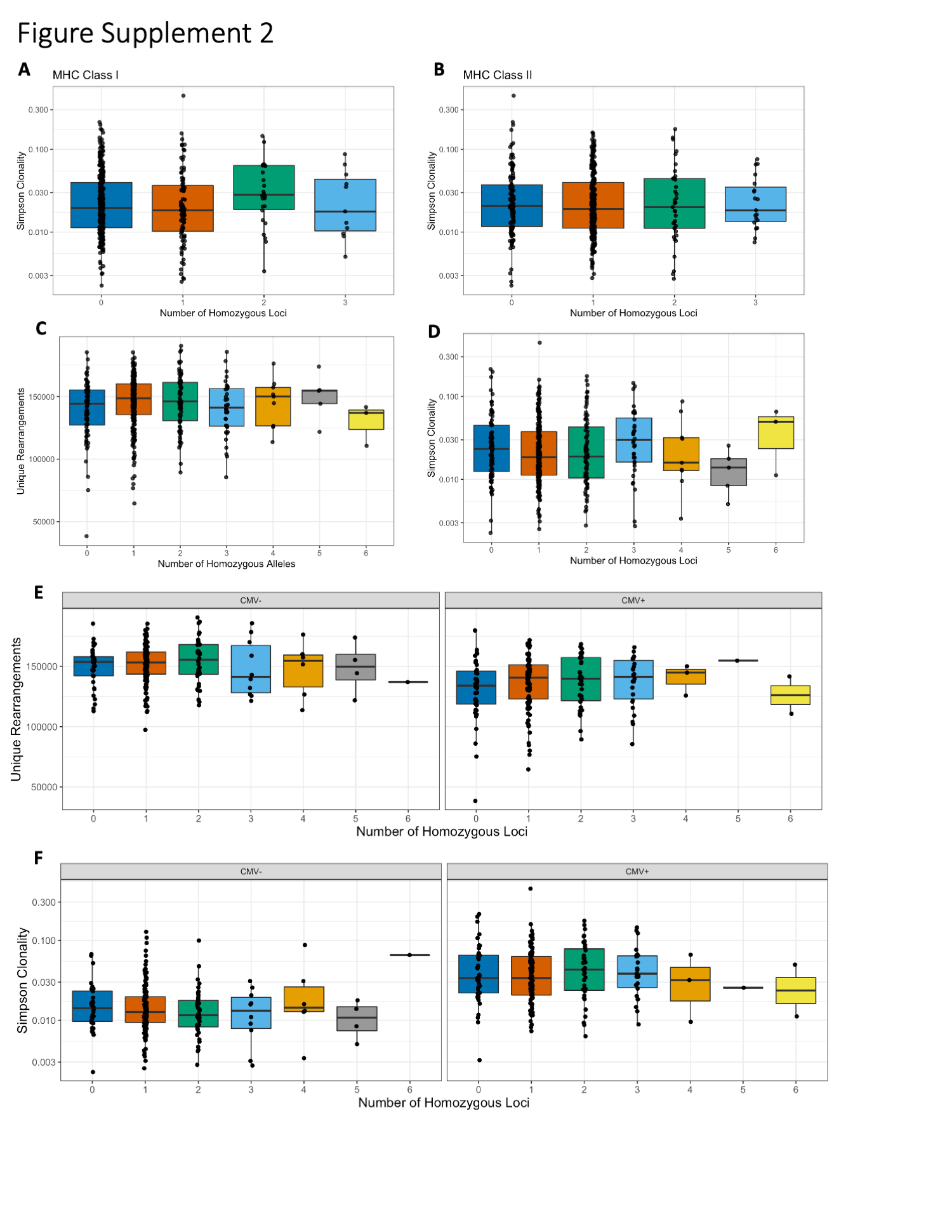

Supplement: S2 Fig — HLA zygosity at neither (A) class I loci (Spearman rho = 0.011, p = 0.08) nor (B) class II loci were correlated with Simpson clonality (-0.023, p = 0.64). Overall, HLA zygosity in this cohort was not correlated with (C) richness (Spearman rho = 0.026, p = 0.60) or (D) Simpson clonality (Spearman rho = 0.0005, p = 0.99). (E) HLA zygosity was not correlated with richness among CMV- individuals (Spearman rho = 0.018, p = 0.79) or CMV+ individuals (Spearman rho = 0.086, p = 0.23). (F) HLA zygosity was not correlated with Simpson clonality among CMV- individuals (Spearman rho = -0.064, p = 0.34) or CMV+ individuals (Spearman rho = 0.023, p = 0.75). (TIF) [file pone.0249484.s002.tif]

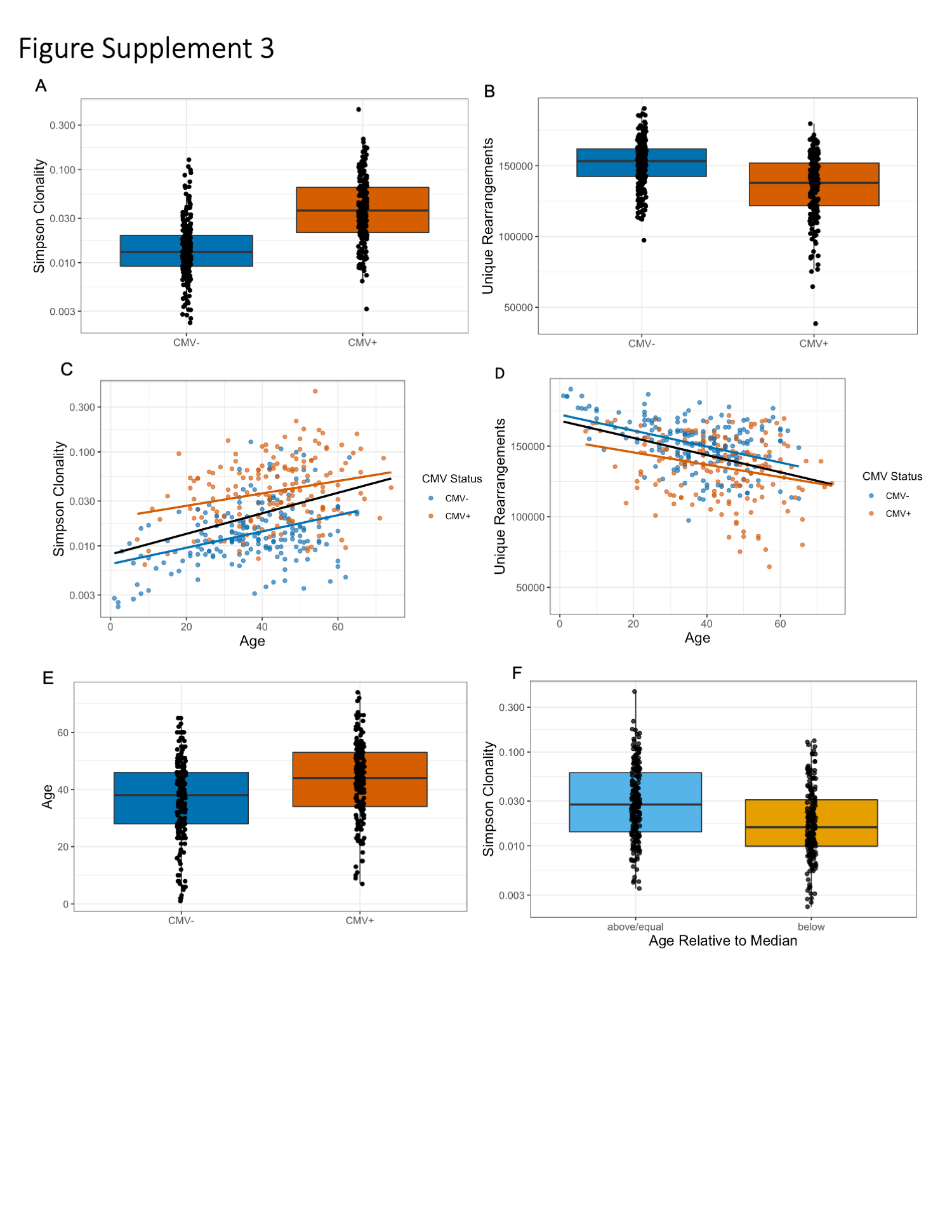

Supplement: S3 Fig — CMV+ Individuals had significantly (A) higher Simpson clonality (Wilcoxon rank sum test, p < 2.2e-16) and (B) lower richness (Wilcoxon rank sum test, p = 4.2e-14) than CMV- individuals. (C) Simpson clonality was positively correlated with age overall (Spearman rho = 0.34, p = 3.4e-11) and within both CMV+ (Spearman rho = 0.23, p = 0.003) and CMV- individuals (Spearman rho = 0.31, p = 1.4e-3). (D) Repertoire richness was inversely correlated with age overall (Spearman rho = -0.35, p = 8.6e-12) and within both CMV+ (Spearman rho = -0.23, p = 0.002) and CMV- individuals (Spearman rho = -0.39, p = 3.4e-8). (E) CMV+ individuals were significantly older than CMV- individuals (Wilcoxon rank sum test, p = 2.9e-5). (F) Subjects older than the overall median age had significantly greater Simpson clonality values than individuals that are younger than or equal to the median age (Wilcoxon rank sum test, p = 2.0e-7). (TIF) [file pone.0249484.s003.tif]

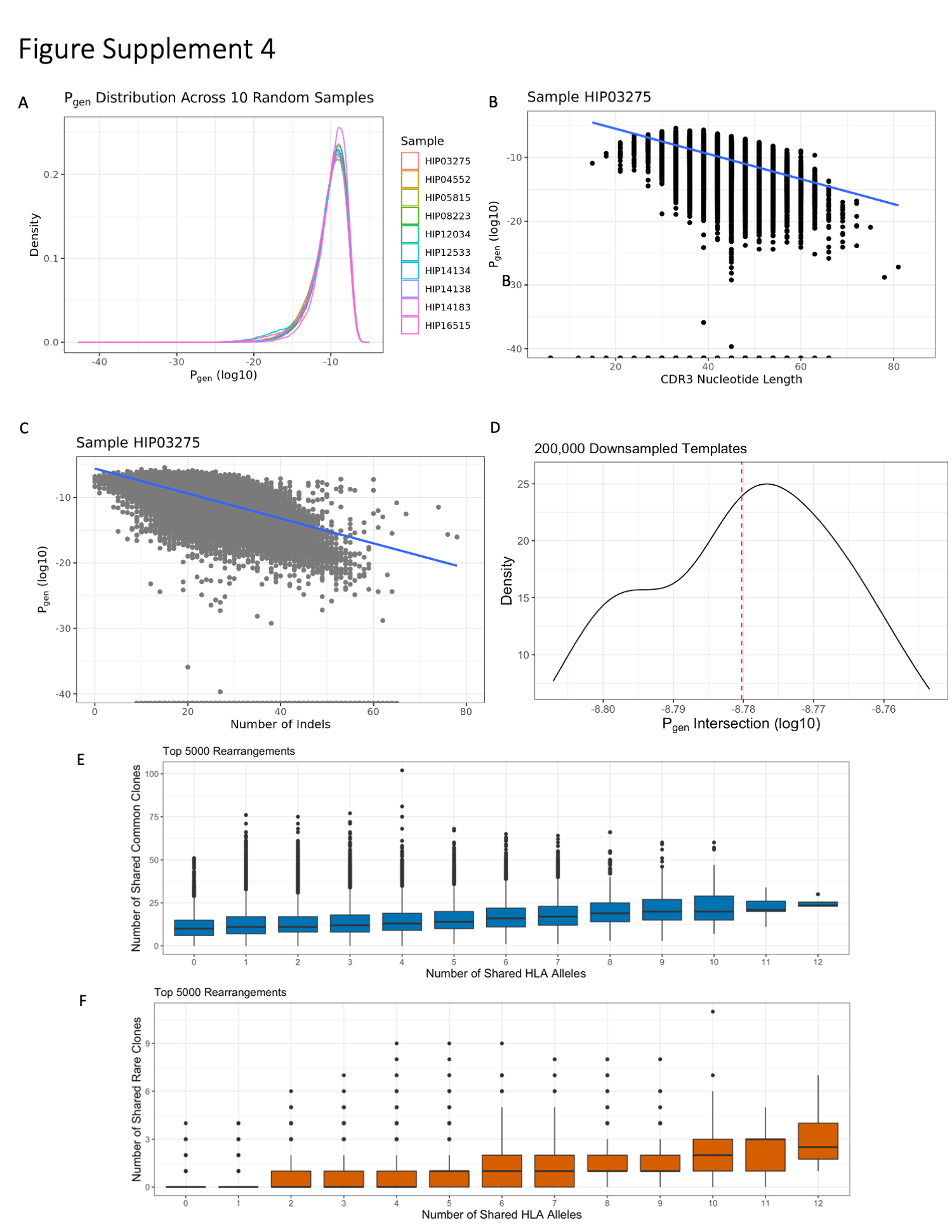

Supplement: S4 Fig — (A) Distribution of generative probabilities of TCRβ clones from 10 representative individuals. Generation probability was significantly correlated with (B) CDR3 nucleotide sequence length (Spearman rho = -0.48, p < 2.2e-16) and (C) number of insertions and deletions (indels) (Spearman rho = -0.63, p < 2.2e-16), figure from single representative repertoire. (D) Identification of a generation probability cutoff point to distinguish between common and rare clones. Generation probability intersection points from the 31 chunks ranged from -8.81 to -8.76 (log10 transformed values) and the median -8.78 (1.66e-9) was selected as the cutoff point. Within the 5000 most abundant rearrangement subset, the number of shared HLA alleles was significantly correlated with clone sharing among both (E) common (Mantel rho = 0.19, p < 1e-3) and (F) rare clones (Mantel rho = 0.28, p < 1e-3). (TIF) [file pone.0249484.s004.tif]

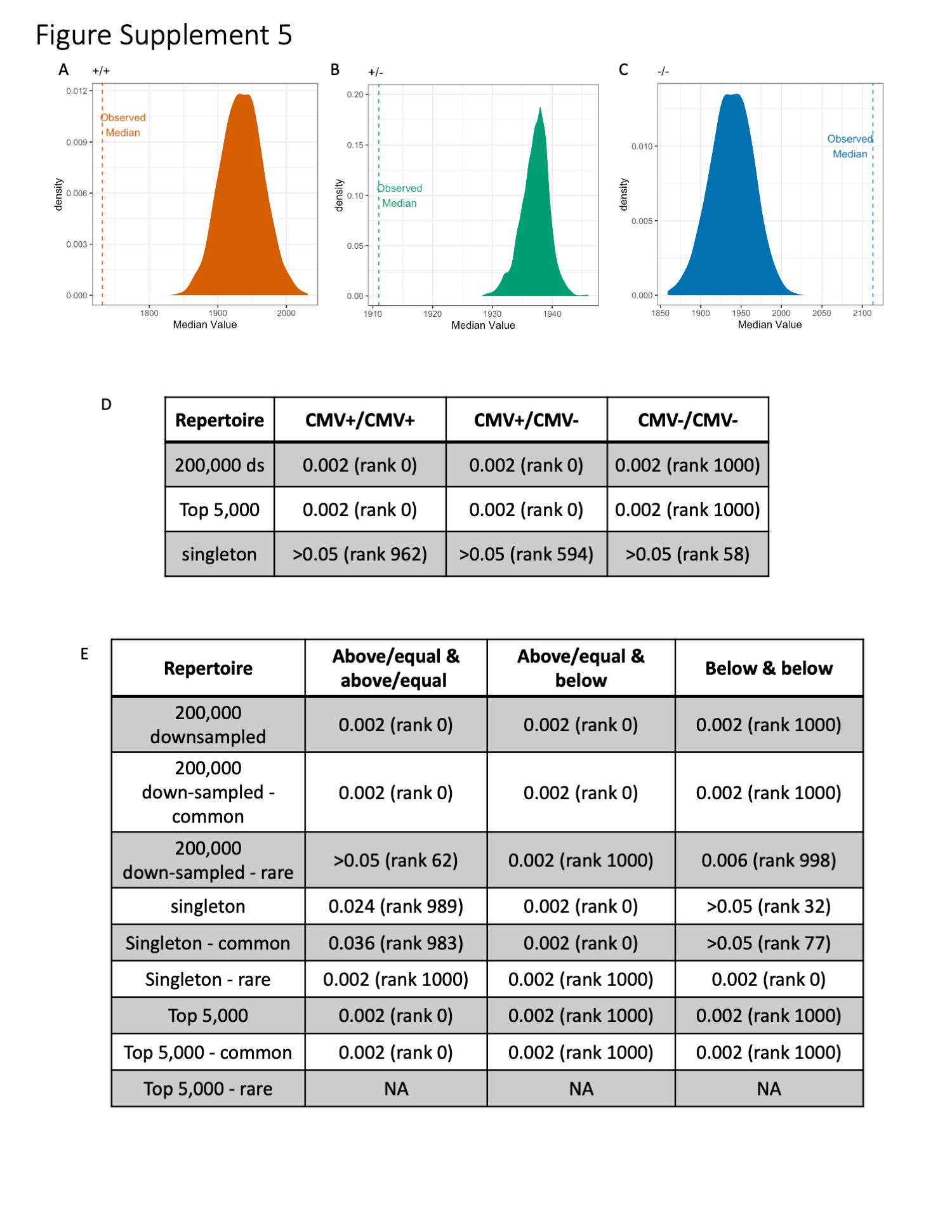

Supplement: S5 Fig — Within each set of pairwise comparisons, we compared the observed median value to the medians of 1,000 permuted comparisons and reported both rank and p-value for distribution indicated. (A) The observed median number of clones shared between CMV+ individuals was lower than the median of all shuffled comparisons, yielding a significant empirical p-value of 0.002. (B) The observed median number of clones shared between CMV+ and CMV- individuals was lower than the median of all shuffled comparisons, yielding a significant empirical p-value of 0.002. (C) The observed median number of clones shared between CMV- individuals was greater than the median of all shuffled comparisons, yielding a significant empirical p-value of 0.002. (D) Table containing the rank and empirical p-values of all clone sharing comparisons stratified by CMV serostatus. (E) Table containing the rank and empirical p-values of all clone sharing comparisons stratified by age relative to median age (42). (TIF) [file pone.0249484.s005.tif]

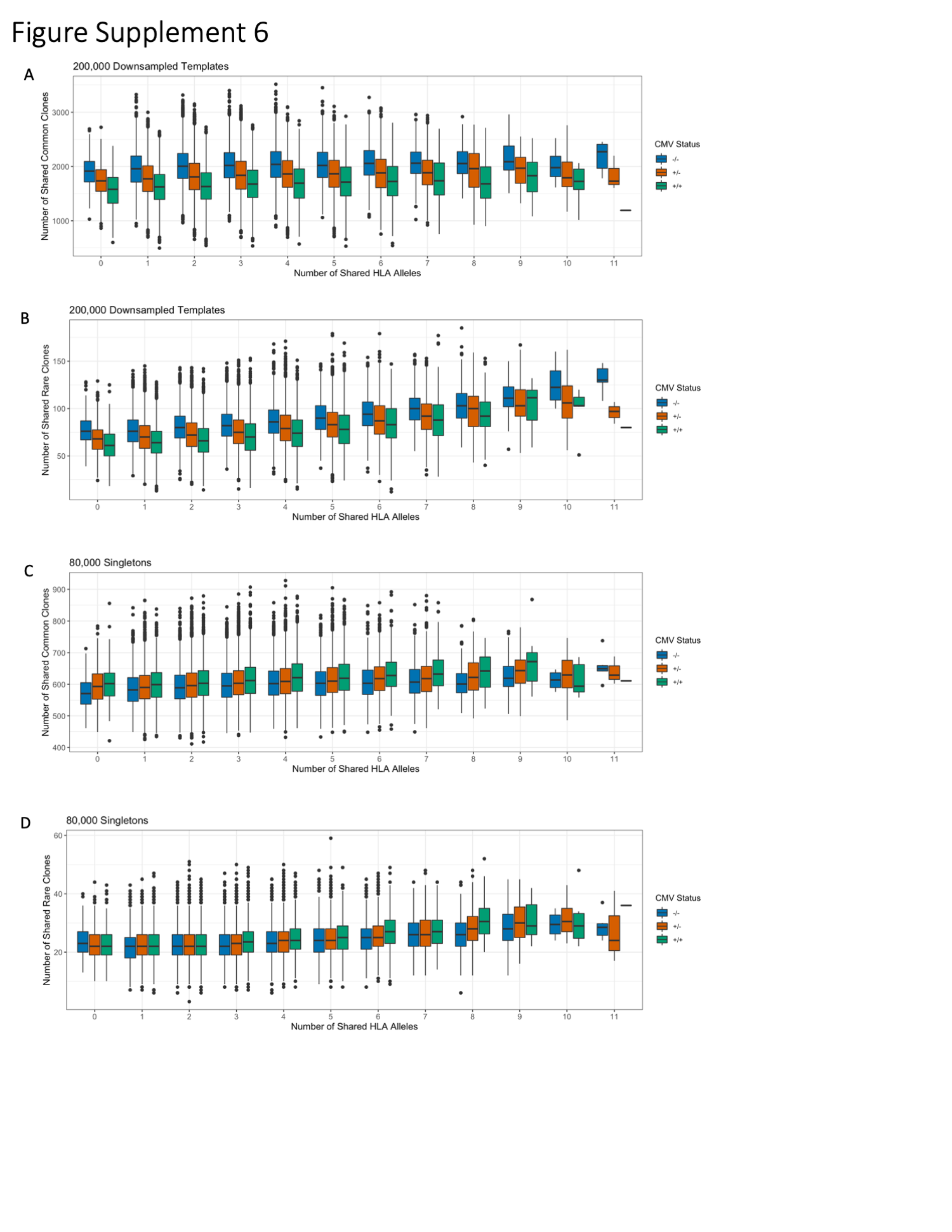

Supplement: S6 Fig — (A) Individuals that were both CMV- shared more common clones than individuals that were both CMV+, regardless of the number of HLA alleles shared. (B) Individuals that were both CMV- shared more rare clones than individuals that were both CMV+, regardless of the number of HLA alleles shared. Individuals that were both CMV+ shared more (C) common and (D) rare singletons than individuals that were both CMV-, regardless of the number of HLA alleles shared. (TIF) [file pone.0249484.s006.tif]

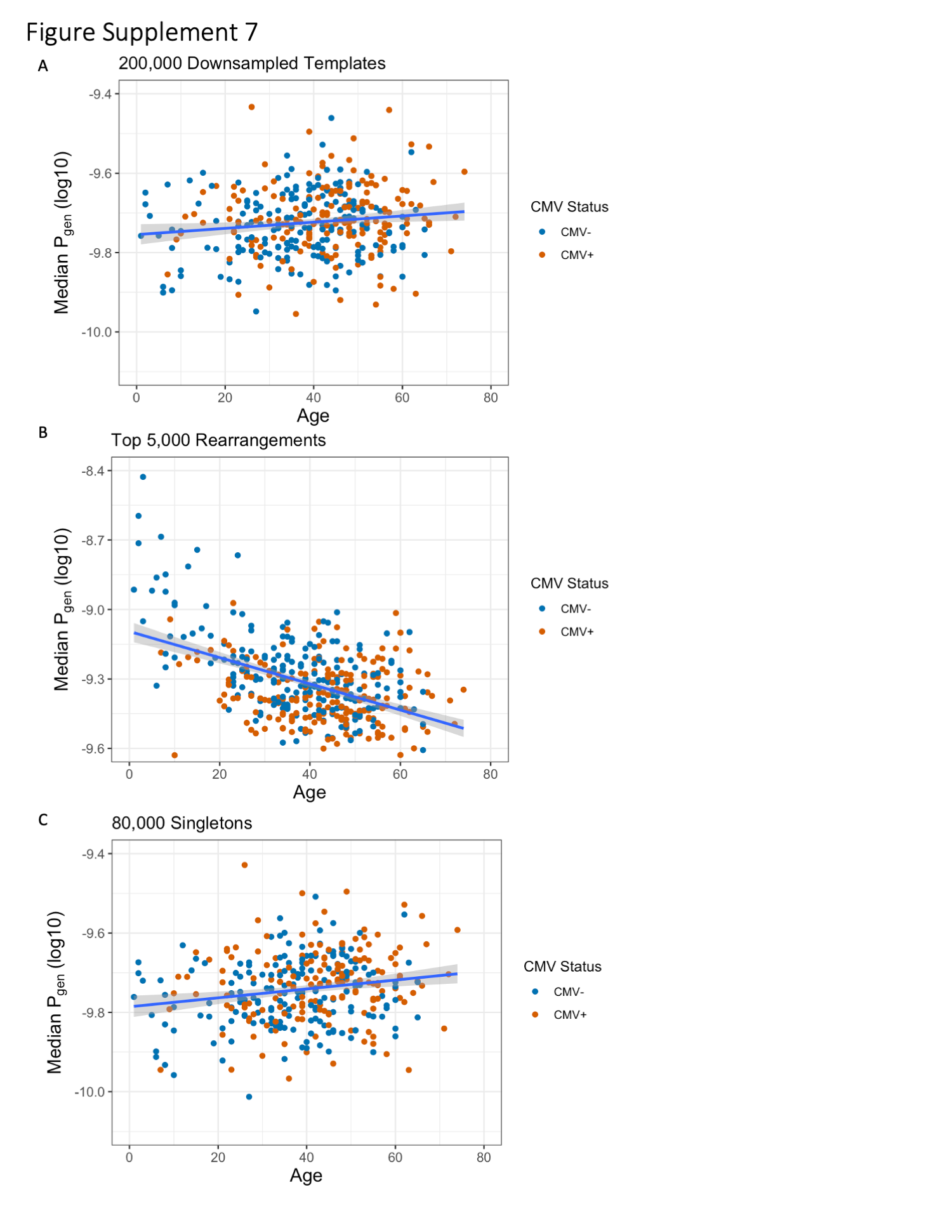

Supplement: S7 Fig — Age was significantly correlated with (A) higher median generation probability within the down-sampled repertoires (Spearman rho = 0.12, p = 0.022), (B) lower median generation probability within the top 5,000 clones (Spearman rho = -0.37, p = 8.6e-15), and (C) higher median generation probability within the singleton repertoires (Spearman rho = 0.17, p = 0.0015). Only subjects with available age data were included in this analysis. (TIF) [file pone.0249484.s007.tif]

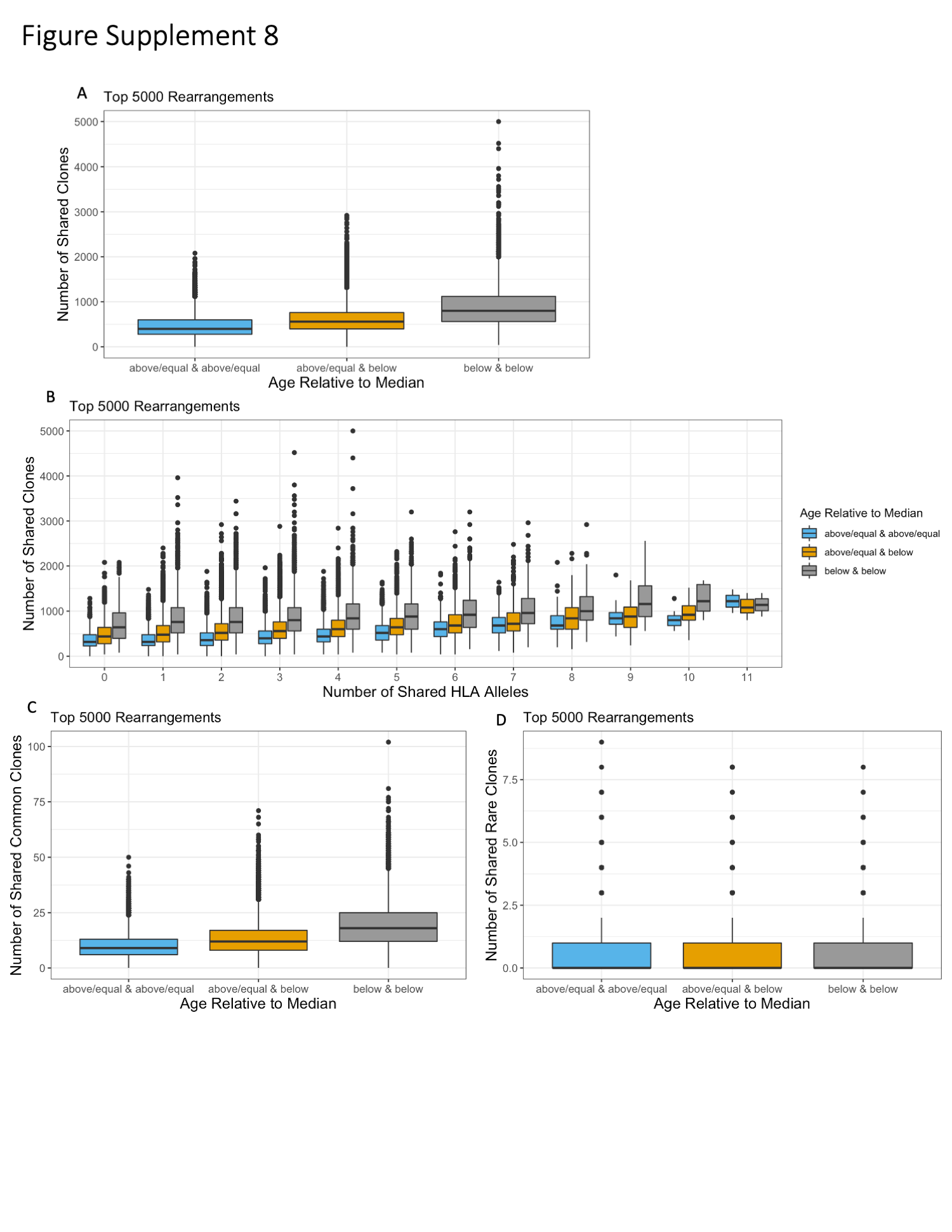

Supplement: S8 Fig — Individuals were stratified by the median age (42). (A) Younger individuals shared more clones than older individuals. (B) Age impacted the sharing of the top 5000 clones regardless of the number of HLA alleles shared. Younger individuals shared more (C) common clones, but no difference between age groups in (D) rare clones sharing was observed. (TIF) [file pone.0249484.s008.tif]

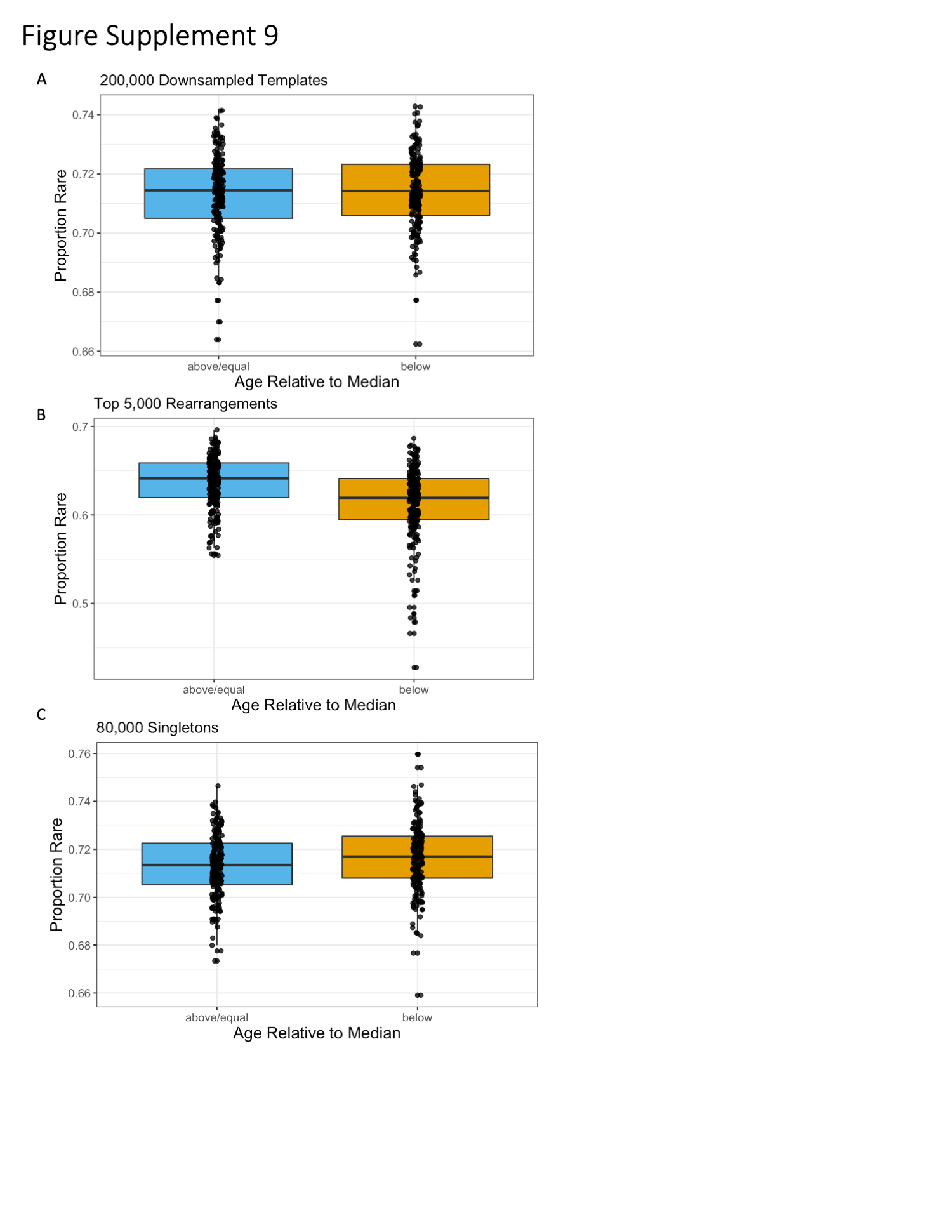

Supplement: S9 Fig — (A) There was not a significant difference in proportion of rare clones between older and younger individuals within the down-sampled repertoires (Wilcoxon rank sum test, p = 0.5). (B) Older individuals had a significantly greater proportion of rare clones among their top 5,000 most abundant rearrangements than younger individuals (Wilcoxon rank sum test, p = 3.6e-10. (C) Older individuals had a significantly lower proportion of rare singletons compared to younger individuals (Wilcoxon rank sum test, p = 3.0e-2). (TIF) [file pone.0249484.s009.tif]
